# Educational inequalities in trajectories of co-occurring behavioral health risk factors over four years: a latent class growth analysis

**DOI:** 10.1186/s12889-025-24663-3

**Published:** 2025-10-02

**Authors:** Sophie Baumann, Andreas Staudt, Maria Zeiser, Jennis Freyer-Adam, Diana Guertler, Henriette Markwart, Anne Moehring, Ulrich John

**Affiliations:** 1 Department of Methods in Community Medicine, Institute for Community Medicine, University Medicine Greifswald, Walther-Rathenau-Str. 48, Greifswald, 17475 Germany; 2 Institute and Policlinic of Occupational and Social Medicine, Faculty of Medicine, Technische Universität Dresden, Fetscherstr. 74, Dresden, 01307 Germany; 3 Department of Prevention Research and Social Medicine, Institute for Community Medicine, University Medicine Greifswald, Walther-Rathenau- Str. 48, Greifswald, 17475 Germany

**Keywords:** Alcohol, Smoking, Overweight, Physical activity, Education, Latent class growth curve analysis, Trajectories, Behavioral health risk factors

## Abstract

**Background:**

This study aimed to identify unobserved subgroups of persons with similar trajectories of co-occurring behavioral health risk factors (BRFs) over 4 years in an adult general population sample and to examine their relation to education.

**Methods:**

Data of 831 control group participants of a randomized controlled trial was analyzed. Participants aged 18–64 years who had consumed alcohol in the past 12 months were recruited at a registry office. Alcohol consumption, tobacco smoking, body mass index, and physical inactivity were assessed at baseline via self-report and 1, 3, and 4 years later. Latent class growth analysis was used to identify BRF trajectories and to test their association with school education, adjusted for sex, age, marital status, and self-rated health.

**Results:**

Eight BRF trajectory classes were identified, with the largest class (31% of participants) being characterized by stable low-to-moderate alcohol consumption and an otherwise healthy lifestyle. Higher-educated compared to lower-educated participants were more likely in this class than in other classes, including, among others, a class combining stable alcohol consumption, tobacco smoking, and increasing excess weight (Odds ratio, OR = 7.41, *p* < 0.001) and a class maintaining absence of BRFs over time (OR = 3.89, *p* < 0.001).

**Conclusions:**

This study demonstrates heterogeneity in BRF trajectories and their relation to education. Although higher-educated persons were more likely in classes with fewer BRFs and favorable BRF changes, alcohol consumption remained a common BRF in several of these trajectories. Trajectories of multiple co-occurring BRFs, particularly those involving tobacco smoking and unfavorable BRF changes, seem to disproportionately affect lower-educated persons.

## Background

There are three potential reasons why alcohol consumption, tobacco smoking, overweight, and physical inactivity are a frequent topic among researchers, practitioners, and the public. First, these behavioral risk factors (BRFs) are highly prevalent in the general population, with the majority of persons reporting two or more BRFs [1, 2]. Second, they are major contributors to morbidity and mortality associated with prevalent chronic diseases, such as cardiovascular diseases and several types of cancer [3–5]. Third, they often interact synergistically to add more to morbidity and mortality risk than expected from either BRF alone [3, 6]. However, there is still a gap in the literature regarding how and why BRFs interact.

A main limitation in current research is that the co-occurrence of BRFs has mostly been viewed as a cross-sectional phenomenon. However, none of the BRFs can be adequately conceptualized as a static entity [7, 8]. For example, smoking initiation has been linked to higher alcohol consumption and vice versa, whereas smoking cessation is often followed by reduced drinking but weight gain [9, 10]. Another critical issue is whether the associations between BRF trajectories are the same among all persons within a population. Previous findings on average changes in BRFs, their determinants and consequences may be misleading, as average associations may obscure a mixture of positive associations for some persons and little, no, or even negative associations for others. For example, if certain groups of persons are more likely than others to smoke tobacco while drinking alcohol, or are less likely to gain weight after quitting smoking, individual BRF trajectories would be clustered. This means that the population is composed of distinct subgroups defined by their trajectory of co-occurring BRFs. It is not possible to directly observe which group a person belongs to. This represents a case of unobserved heterogeneity.

Longitudinal mixture models are useful to better understand linked changes across BRFs by identifying unobserved groups of persons with similar trajectories of co-occurring BRFs. Studies utilizing such approaches have often been focused on specific stages in life, such as the transition from adolescence to adulthood or from high school to college [11–13]. Only a few studies have examined the heterogeneity in BRF trajectories in the general population. A study of U.S. adults aged 25 years or older found five distinct BRF profiles, with the most prevalent profile being a healthy lifestyle, except for low-to-moderate alcohol consumption [14]. Most profiles were quite stable over time, except for current smoking. Another study of U.S. adults aged 30 years or older found three BRF profiles labelled healthy, low energy balance, and unhealthy, with most groups of persons remaining in or transitioning to the healthy BRF profile over time [15].

Another important issue to consider is that trajectories of co-occurrent BRFs may vary by socio-economic position. The health lifestyle theory predicts that BRFs would accumulate in groups of persons with lower socio-economic position as their social class position may limit the set of health choices available to them, and in some social contexts, unhealthy behaviors may be more common and socially accepted [16]. While it is well-documented that BRFs tend to co-occur in groups of persons with lower socio-economic position [17], the socio-economic variation in temporal changes in the co-occurrence of these factors remains poorly understood.

The aim of this study was first, to identify distinct groups of persons with similar trajectories of multiple co-occurring BRFs over a four-year period in an adult general population sample and, second, to examine associations of the trajectories with education as an indicator of socio-economic position.

## Methods

### Design

This study presents control group data from the randomized controlled trial entitled “Testing a proactive expert system intervention to prevent and to quit at-risk alcohol use” (PRINT), which aimed to test the 12-month efficacy of computer-generated individualized feedback [18]. The trial was prospectively registered at the German Clinical Trials Register (trial identifier: DRKS00014274, date of registration: 12 March 2018) and approved by the ethics committee of University Medicine Greifswald (BB 147/15). The participants provided written informed consent. Renewal funding was obtained for 3- and 4-year follow-ups, with approval by the ethics committees of University Medicine Greifswald (BB 053/19) and Technische Universität Dresden (SR-EK-272062020).

### Participants

Participants were proactively recruited at a registry office in Greifswald, a city of approximately 60,000 residents in Mecklenburg-Western Pomerania, Germany, from April to June 2018. Study assistants invited all persons aged 18–64 years appearing in the waiting area to complete a self-administered survey on a tablet computer, which included an eligibility screening for the trial. Persons who had already been approached, those with cognitive impairments or physical conditions preventing trial participation, those with insufficient German language or reading skills, or those employed at the conducting research institute were excluded. Persons who had consumed alcohol in the previous 12 months were eligible for trial inclusion. Those without a telephone or permanent address were excluded. All participants received a self-selected 5-euro voucher.

As described in more detail elsewhere [19], 1646 persons (67% of eligibles) participated in the trial. For this study, only data from the 831 participants randomized to the control group were used. The control group received assessment only at baseline, 3 months, and 6 months, but no intervention.

### Follow-Ups

One-, 3-, and 4-year follow-ups were conducted via structured computer-assisted telephone interviews by trained study staff in April to July 2019, 2021, and 2022, respectively. Ten telephone contact attempts were made before sending a questionnaire by email or postal mail, with up to two reminders. Participants received a 5-euro voucher prior to each assessment.

As described elsewhere [18, 20], of the 831 control group participants, 676 (81%) participated in the 1-year follow-up, 555 (65%) in the 3-year follow-up, and 504 (61%) in the 4-year follow-up. Logistic regression analyses showed higher follow-up participation among older compared to younger persons (p-values < 0.01), persons with ≥ 12 years of schooling compared to < 12 years (p-values < 0.001), persons with normal weight compared to those with overweight (p-values < 0.05), current non-smokers compared to less than daily (p-values < 0.01) or daily smokers (p-values < 0.001). Sex, self-rated health, alcohol consumption, and physical inactivity were not significantly associated with follow-up participation.

### Measures

Alcohol consumption was calculated based on self-reports of frequency and quantity of drinking in the past 30 days: „How often did you have an alcoholic drink: never [*frequency multiplier: 0 drinking days per month*], once [*1*], 2–4 times [*3*], 2–3 times/week [*10*], or ≥ 4 times/week [*22*]?“ and „How many drinks did you typically have on a drinking day?“. A drink was defined as 10 grams of pure alcohol equivalent to 0.25–0.3 l beer, 0.1–0.15 l wine or sparkling wine, or 4 cl spirits. To determine drinks/week, frequency was multiplied by the number of drinks, and the total was divided by the number of weeks in a month. Alcohol consumption was then categorized into currently abstinent (0 drinks in the past 30 days), low-to-moderate (women: 0.1–7 drinks/week, men: 0.1–14 drinks/weeks), and high (women: >7 drinks/week, men: >14 drinks/week). Additionally, alcohol consumption was categorized as high if participants reported either heavy episodic drinking (women: ≥ 4 drinks/occasion, men: ≥ 5 drinks/occasion) at least once a month in the third question of the Alcohol Use Disorders Identification Test [21], or having a heavy drinking day (women: ≥ 4 drinks, men: ≥ 5 drinks) on any day in the past week, as assessed by the timeline follow-back method [22].

Tobacco smoking was assessed with the question „Do you smoke currently?“ and four possible responses: „Yes, I smoke daily“ (current daily smoking), „Yes, I smoke sometimes“ (current less than daily smoking), „No, I do not smoke anymore“ (former smoking), and „No, I have never smoked“ (never smoking). Former and never smoking were merged to non-smoking.

The body mass index was obtained from self-reported body weight in kilogram and body height in meters and the formula weight divided by height squared. The body mass index was categorized into normal weight (< 25 kg/m^2^), overweight (25–29.9 kg/m^2^), and obesity (≥ 30 kg/m^2^).

Physical activity was assessed by asking the participants how many minutes they spent on moderate-to-vigorous physical activity (MVPA) during leisure time on a typical weekday and a typical weekend day, respectively. MVPA was defined as activities that increase heart rate and breathing, such as fast walking, jogging, cycling, swimming, or gardening. Responses were translated to minutes/week by summing the weekday time multiplied by 5 and the weekend time multiplied by 2. Finally, MVPA was categorized into < 150 min/week, 150–300 min/week, and > 300 min/week.

Education was assessed at baseline by asking participants to indicate their highest general educational degree. The response options included an exhaustive list of possible school-leaving qualifications in Germany and equivalent foreign degrees. The information was categorized into ≤ 9 years, 10–11 years, and ≥ 12 years of schooling. The first two categories were merged due to the limited number of participants in each category.

Other covariates were assessed at baseline and included sex (male, female), age, and marital status (single, married, separated/divorced/widowed). Self-rated health was assessed with the question „Would you say your health in general is: excellent, very good, good, fair, or poor?“ [23]. Fair and poor were merged due to the limited number of participants in each of the two categories.

### Statistical analysis

To identify unobserved subgroups of persons with similar trajectories of co-occurring BRFs over time, a parallel process latent class growth analysis (LCGA) was conducted using Mplus version 8.8 [24]. Although most applications of this methodology have focused on modeling two longitudinal outcomes simultaneously [25], parallel process models for more than two outcomes have been successfully applied in behavioral health research [12, 13, 26]. Trajectory was defined as the developmental course of alcohol consumption, tobacco smoking, body mass index, or physical inactivity from baseline to 4-year follow-up. Each trajectory may have changed or remained stable over time.

For each of the four BRFs, the trajectory was captured by latent growth factors representing the initial level (intercept) and the rate of change (slope). These factors were based on four categorical indicators representing the respective BRF at each time point. Time scores were treated as parameters to be estimated to capture non-linear trajectories. The growth factors of the four BRFs were regressed on a categorical latent variable representing the unobserved subgroups (latent classes) with similar trajectories of co-occurring BRFs over time. In other words, classes were defined by their trajectories of alcohol consumption, tobacco smoking, body mass index, and physical inactivity over 4 years.

A full-information maximum likelihood (FIML) estimator with robust standard errors was used to estimate the models. With FIML, all available data in the variance-covariance matrix are used to find the model parameters that maximizes the likelihood for the observed data. This approach enables the inclusion of all 831 control group participants in the analysis, even those who missed later follow-ups. FIML represents a best-practice approach for handling missing data in longitudinal latent variable modeling, providing unbiased parameter estimates under the assumption that data are missing at random (MAR) assumption [27, 28]. To avoid local solutions, 1,000 sets of random starting values with 10 final stage optimizations were used. The number of initial stage iterations was set to 50 and the maximum number of iterations for final optimization was set to 1,000.

The optimal number of classes was determined using information criteria, theoretical interpretability, and class size. Akaike information criterion (AIC), Bayesian information criterion (BIC), and sample-size adjusted BIC were reported, with lower values indicating better fit [29–31]. The Vuong-Lo-Mendell-Rubin adjusted likelihood ratio test (VLMR-LRT) was used to compare the estimated model with a model with one class less. P-values < 0.5 indicated that the model with one class less does not fit the data as well as the estimated model. Classification diagnostics were examined, but not used for model selection. Entropy values and average conditional class probabilities of correct class-classification were obtained, with values close to 1 indicating clear classification [32, 33]. For each class in the selected optimal class solution, the number and proportion of participants allocated to this class as well as the probabilities of observing a certain BRF for each time point were presented. Probability values can range between 0 and 1, with higher values indicating a higher probability of observing a certain BRF.

Education, sex, age, marital status, and self-rated health were specified as auxiliary variables in order to use them as predictors of class membership in a three-step approach [34]. This approach retained the classification of the final unconditional LCGA model while accounting for classification uncertainty when estimating the relationship between education and BRF trajectory class, adjusted for the other covariates in a multinomial logistic regression. Odds ratios (ORs) with 95% confidence intervals (CIs) were calculated. In addition to this, significant differences after Bonferroni-Holm correction for multiple comparisons were reported. ORs of 1.44, 2.48, and 4.27 (and their reciprocals: 0.70, 0.40, and 0.23) were used as benchmarks for small, medium, and large effects, respectively, corresponding to Cohen’s d effect sizes of ± 0.2, ± 0.5, and ± 0.8. The transformation of d in ORs was done according to Borenstein et al. [35] using a calculator provided by Lenhard and Lenhard [36].

## Results

### Sample description

The sample (*n* = 831) was composed of 460 females (55.4%) and 371 males (44.6%), with a mean age of 30.8 years (SD = 10.8). About two-thirds of the participants had ≥ 12 years of schooling (*n* = 557, 67.0%). At baseline, most participants reported low-to-moderate alcohol consumption or current abstinence (*n* = 554, 66.7%), being a non-smoker (*n* = 550, 66.2%), having a normal weight (540, 65.0%), and engaging in > 300 min/week of MVPA (*n* = 483, 58.1%). Observed categories of the BRFs at the four time points are depicted in Table 1.

**Table 1:** Numbers and percentages of participants with a certain behavior-related health risk factor at baseline and follow-ups

	Total sample (*n* = 831)				≤ 11 years of schooling (*n* = 274)				≥ 12 years of schooling (*n* = 557)			
	Baseline	Year 1	Year 3	Year 4	Baseline	Year 1	Year 3	Year 4	Baseline	Year 1	Year 3	Year 4
Alcohol consumption												
Currently abstinent	76 (9.2)	56 (8.3)	48 (8.7)	41 (8.1)	35 (12.8)	21 (10.8)	16 (11.3)	11 (9.0)	41 (7.3)	35 (7.3)	32 (7.8)	30 (7.9)
Low-to-moderate	478 (57.5)	419 (62.0)	354 (63.9)	315 (62.5)	159 (58.0)	130 (66.7)	92 (64.8)	77 (63.1)	319 (57.3)	289 (60.1)	262 (63.6)	238 (62.3)
High	277 (33.3)	201 (29.7)	152 (27.4)	148 (29.4)	80 (29.2)	44 (22.5)	34 (23.9)	34 (27.9)	197 (35.4)	157 (32.6)	118 (28.6)	114 (29.8)
Wald Χ^2^ test	Χ^2^ (2) = 6.5, *p* = 0.040				Χ^2^ (2) = 2.0, *p* = 0.367				Χ^2^ (2) = 7.4, *p* = 0.024			
Tobacco smoking												
Non-smoking	550 (66.2)	507 (75.0)	433 (78.0)	408 (81.0)	132 (48.2)	108 (55.4)	95 (66.4)	89 (73.0)	418 (75.1)	399 (83.0)	338 (82.0)	319 (83.5)
Occasional	92 (11.1)	45 (6.7)	32 (5.8)	31 (6.1)	25 (9.1)	17 (8.7)	5 (3.5)	6 (4.9)	67 (12.0)	28 (5.8)	27 (6.6)	25 (6.5)
Daily	189 (22.7)	124 (18.3)	90 (16.2)	65 (12.9)	117 (42.7)	70 (35.9)	43 (30.1)	27 (22.1)	72 (12.9)	54 (11.2)	47 (11.4)	38 (10.0)
Wald Χ^2^ test	Χ^2^ (2) = 53.2, *p* < 0.001				Χ^2^ (2) = 39.7, *p* < 0.001				Χ^2^ (2) = 12.8, *p* = 0.002			
Body mass index												
Normal weight	540 (65.0)	435 (64.4)	328 (59.2)	311 (61.7)	139 (50.7)	98 (50.3)	57 (40.1)	50 (41.0)	401 (72.0)	337 (70.1)	271 (65.8)	261 (68.3)
Overweight	213 (25.6)	169 (25.0)	164 (29.6)	136 (27.0)	96 (35.0)	64 (32.8)	58 (40.9)	46 (37.7)	117 (21.0)	105 (21.8)	106 (25.7)	90 (23.6)
Obesity	78 (9.4)	72 (10.6)	62 (11.2)	57 (11.3)	39 (14.2)	33 (16.9)	27 (19.0)	26 (21.3)	39 (7.0)	39 (8.1)	35 (8.5)	31 (8.1)
Wald Χ^2^ test	Χ^2^ (2) = 8.5, *p* = 0.014				Χ^2^ (2) = 11.6, *p* = 0.003				Χ^2^ (2) = 7.0, *p* = 0.031			
Physical activity												
< 150 min/week	114 (13.7)	49 (7.2)	60 (10.8)	38 (7.5)	38 (13.9)	15 (7.7)	20 (14.1)	9 (7.4)	76 (13.6)	34 (7.1)	40 (9.7)	29 (7.6)
150–300 min/week	234 (28.2)	150 (22.2)	117 (21.1)	103 (20.4)	48 (17.5)	45 (23.1)	24 (16.9)	20 (16.4)	186 (33.4)	105 (21.8)	93 (22.6)	83 (21.7)
> 300 min/week	483 (58.1)	477 (70.6)	377 (68.1)	363 (72.0)	188 (68.6)	135 (69.2)	98 (69.0)	93 (76.2)	295 (53.0)	342 (71.1)	279 (67.7)	270 (70.7)
Wald Χ^2^ test	Χ^2^ (2) = 27.0, *p* < 0.001				Χ^2^ (2) = 2.0, *p* = 0.366				Χ^2^ (2) = 30.3, *p* < 0.001			

Data are numbers (%). Wald Χ2 statistics from multinomial logistic regression models with cluster-robust standard errors to account for within-subject correlations.

### BRF trajectory classes

Model fit and diagnostic criteria for alternative class solutions are shown in Table 2. The VLMR-LRT indicated that the 8-class model was preferable. Although the smallest class included only 27 participants, all classes were considered qualitatively distinct and meaningful. Therefore, the 8-class model was chosen for further analysis. Entropy was 0.88 and average latent class posterior probabilities ranged from 0.81 to 1.00.

**Table 2:** Model fit and diagnostic criteria for alternative latent class growth models

	Model fit criteria					Diagnostic criteria		
Models	LL (# free parameters)	AIC	BIC	aBIC	VLMR-LRT	Smallest class, *n* (%)	Entropy	ALCPP
1 class	−8503 (20)	17,047	17,141	17,077	—	831 (100.0)	—	—
2 classes	−7857 (29)	15,773	15,910	15,818	< 0.001	296 (35.6)	0.90	0.97–0.98
3 classes	−7446 (38)	14,968	15,147	15,027	< 0.001	76 (9.1)	0.92	0.97–1.00
4 classes	−7083 (47)	14,261	14,483	14,334	< 0.001	76 (9.1)	0.91	0.97–1.00
5 classes	−6941 (56)	13,993	14,258	14,080	< 0.001	66 (7.9)	0.90	0.92–1.00
6 classes	−6807 (65)	13,743	14,050	13,844	< 0.001	27 (3.2)	0.91	0.93–1.00
7 classes	−6075 (74)	13,559	13,908	13,673	0.014	27 (3.2)	0.88	0.82–1.00
8 classes	−6613 (83)	13,392	13,784	13,520	0.029	27 (3.2)	0.88	0.81–1.00
9 classes	−6548 (92)	13,280	13,715	13,423	0.324	18 (2.2)	0.89	0.81–1.00
10 classes	−6493 (101)	13,189	13,666	13,345	0.082	18 (2.2)	0.88	0.79–1.00

*LL* log-likelihood, *AIC* Akaike information criterion, *BIC* Bayesian information criterion, *aBIC* sample-size adjusted BIC, *VLMR-LRT* Vuong-Lo-Mendell-Rubin adjusted likelihood ratio test (p-value), *ALCPP* average latent class posterior probability.

**Fig. 1:**
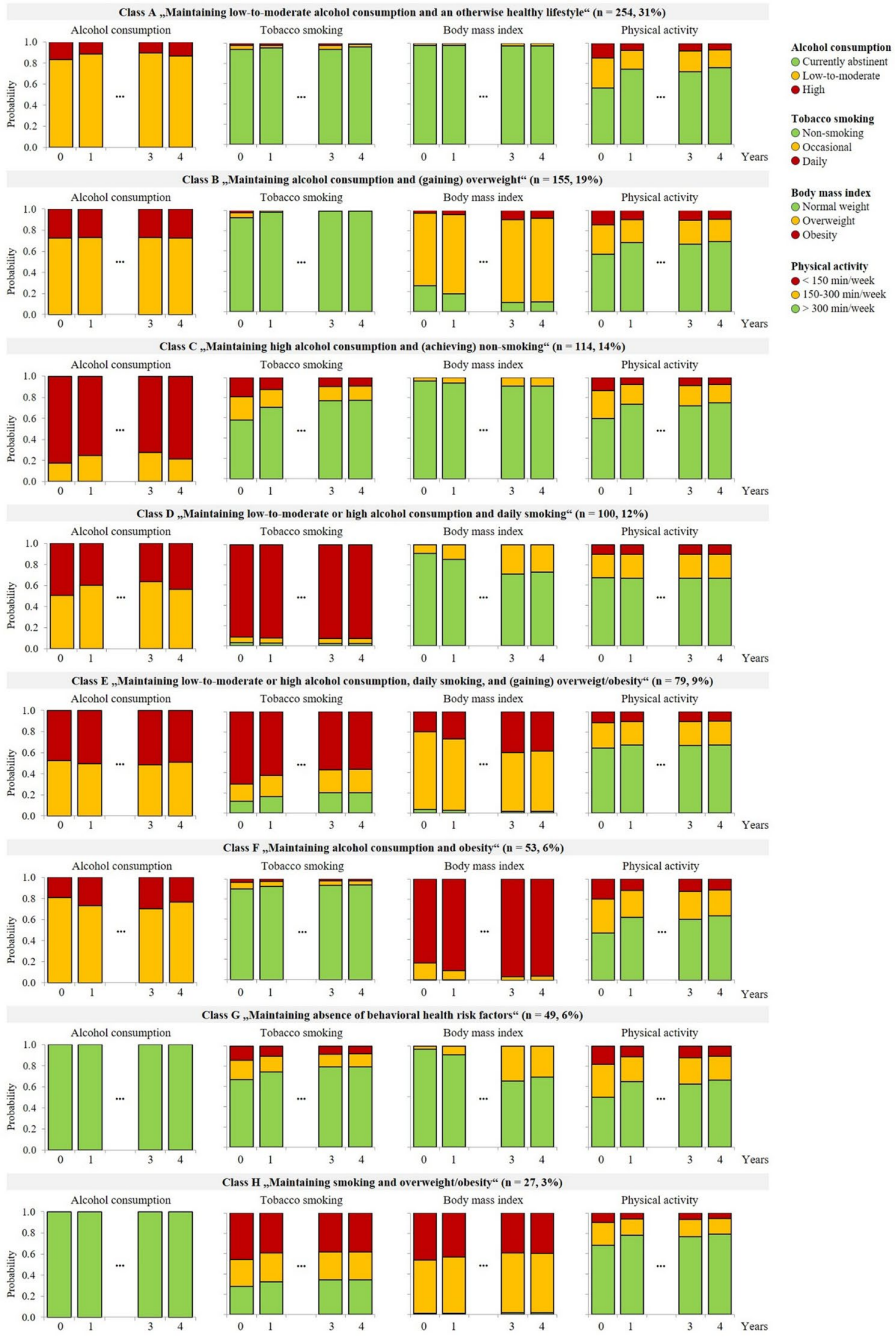
Probabilities of observing a certain behavioral health risk factor over 4 years within each class

As shown in Fig. 1, participants in class A (*n* = 254, 31%) exhibited consistently high probabilities of low-to-moderate alcohol consumption (probability range, pr: 0.83–0.88), non-smoking (pr: 0.94–0.96), and normal weight (pr: 0.97–0.98) across all time points. They also showed moderate but increasing probabilities of engaging in > 300 min/week of MVPA (pr: 0.56–0.76). Class A was labeled „*Maintaining low-to-moderate alcohol consumption and an otherwise healthy lifestyle*“.

Participants in class B (*n* = 155, 19%) consistently consumed alcohol with moderate probabilities of low-to-moderate use (pr: 0.72–0.73). They had consistently high probabilities of non-smoking (pr: 0.93–0.99), high and slightly increasing probabilities of overweight (pr: 0.72–0.83), and moderate and increasing probabilities of engaging in > 300 min/week of MVPA (pr: 0.56–0.69). Class B was labeled „*Maintaining alcohol consumption and (gaining) overweight*“.

Participants in class C (*n* = 114, 14%) consistently consumed alcohol with high probabilities of high use (pr: 0.72–0.82). They had moderate but increasing probabilities of non-smoking (pr: 0.58–0.77), consistently high probabilities of normal weight (pr: 0.91–0.96), and moderate but increasing probabilities of engaging in > 300 min/week of MVPA (pr: 0.59–0.75). Class C was labeled „*Maintaining high alcohol consumption and (achieving) non-smoking*“.

Participants in class D (*n* = 100, 12%) consistently consumed alcohol either at a low-to-moderate (pr: 0.50–0.64) or high level (pr: 0.36–0.50). They had consistently high probabilities of daily smoking (pr: 0.91–0.93), high but decreasing probabilities of normal weight (pr: 0.71–0.91), and a probability of 0.67 of engaging in > 300 min/week of MVPA across all time points. Class D was labeled „*Maintaining low-to-moderate or high alcohol consumption and daily smoking*“.

Participants in class E (*n* = 79, 9%) consistently consumed alcohol either at a low-to-moderate (pr: 0.49–0.52) or high level (pr: 0.48–0.51). They had high but decreasing probabilities of daily smoking (pr: 0.57–0.71), high probabilities of overweight or obesity (pr: 0.96–0.99) with increasing probabilities of obesity (pr: 0.20–0.40), and consistently moderate probabilities of engaging in > 300 min/week of MVPA (pr: 0.64–0.67). Class E was labeled „*Maintaining low-to-moderate or high alcohol consumption*,* daily smoking*,* and (gaining) overweight/obesity*“.

Participants in class F (*n* = 53, 6%) consistently consumed alcohol with moderate to high probabilities of low-to-moderate use (pr: 0.70–0.81). They had consistently high probabilities of non-smoking (pr: 0.90–0.94), high and increasing probabilities of obesity (pr: 0.83–0.97), and moderate and increasing probabilities of engaging in > 300 min/week of MVPA (pr: 0.47–0.64). Class F was labeled „*Maintaining alcohol consumption and obesity*“.

Participants in class G (*n* = 49, 6%) had a probability of 1.0 for abstaining from alcohol in the past 30 days across all time points, moderate but increasing probabilities of non-smoking (pr: 0.67–0.79), high but slightly decreasing probabilities of normal weight (pr: 0.86–1.00), and moderate but increasing probabilities of engaging in > 300 min/week of MVPA (pr: 0.49–0.66). Class G was labeled „*Maintaining absence of BRFs*“.

Participant in class H (*n* = 27, 3%) had a probability of 1.0 for abstaining from alcohol across all time points, consistently low probabilities of non-smoking (pr: 0.28–0.34), a probability of 0.99 for overweight or obesity across all time points, and moderate to high probabilities of engaging in > 300 min/week of MVPA (pr: 0.68–0.79). Class H was labeled „*Maintaining smoking and overweight/obesity*“. Table 3 provides sample characteristics by BRF trajectory class.

**Table 3:** Baseline sample characteristics by latent trajectory class

Characteristics	Total sample	Class A	Class B	Class C	Class D	Class E	Class F	Class G	Class H	Class comparisons^b^
Age in years, M (SD)	30.8 (10.8)	29.8 (9.9)	34.0 (11.5)	25.4 (8.8)	30.1 (10.4)	32.5 (9.9)	39.0 (11.8)	28.6 (9.6)	31.1 (11.2)	*F*(7,823) = 12.4, *p* < 0.001
Sex, n (%)										Χ^2^(7) = 24.6, *p* = 0.001
Female	460 (55.4)	168 (66.1)	73 (47.1)	60 (52.6)	50 (50.0)	35 (44.3)	26 (49.1)	32 (65.3)	16 (59.3)	
Male	371 (44.6)	86 (33.9)	82 (52.9)	54 (47.4)	50 (50.0)	44 (55.7)	27 (50.9)	17 (34.7)	11 (40.7)	
Education, n (%)										Χ^2^(7) = 112.3, *p* < 0.001
< 12 years of schooling	274 (33.0)	42 (16.5)	46 (29.7)	20 (17.5)	57 (57.0)	47 (59.5)	27 (50.9)	19 (38.8)	16 (59.3)	
≥ 12 years of schooling	557 (67.0)	212 (83.5)	109 (70.3)	94 (82.5)	43 (43.0)	32 (40.5)	26 (49.1)	30 (61.2)	11 (40.7)	
Marital status, n (%)										Χ^2^(14) = 40.3, *p* < 0.001
Single	603 (72.5)	183 (77.1)	97 (62.6)	101 (88.6)	80 (80.0)	53 (67.1)	31 (58.5)	38 (77.6)	20 (74.1)	
Married	166 (20.0)	57 (22.4)	41 (26.4)	10 (8.8)	11 (11.0)	19 (24.0)	17 (32.1)	8 (16.3)	3 (11.1)	
Other^a^	62 (7.5)	14 (5.5)	17 (11.0)	3 (2.6)	9 (9.0)	7 (8.9)	5 (9.4)	3 (6.1)	4 (14.8)	
Self-rated health, n (%)										Χ^2^(21) = 90.7, *p* < 0.001
Excellent	66 (8.0)	25 (9.8)	12 (7.7)	13 (11.4)	6 (6.0)	3 (3.8)	1 (1.9)	5 (10.2)	1 (3.7)	
Very good	321 (38.6)	132 (52.0)	55 (35.5)	54 (47.4)	29 (29.0)	17 (21.5)	9 (17.0)	17 (34.7)	8 (29.6)	
Good	384 (46.2)	88 (34.7)	80 (51.6)	42 (36.8)	55 (55.0)	48 (60.8)	33 (62.2)	26 (53.1)	12 (44.4)	
Fair/poor	60 (7.2)	9 (3.5)	8 (5.2)	5 (4.4)	10 (10.0)	11 (13.9)	10 (18.9)	1 (2.0)	6 (22.2)	

*Class A* – Maintaining low-to-moderate alcohol consumption and an otherwise healthy lifestyle

*Class B – *Maintaining alcohol consumption and (gaining) overweight

*Class C – *Maintaining high alcohol consumption and (achieving) non-smoking

*Class D – *Maintaining low-to-moderate or high alcohol consumption and daily smoking

*Class E – *Maintaining low-to-moderate or high alcohol consumption, daily smoking, and (gaining) overweight/obesity

*Class F – *Maintaining alcohol consumption and obesity

*Class G – *Maintaining absence of BRFs

*Class H – *Maintaining smoking and overweight/obesity

*M* mean, *SD* standard deviation.

^a^ Other; separated/divorced/widowed. ^b^ Analysis of variances for continuous variables and chi-square tests for categorical variables.

### Education

Table 4 presents the results of a multinomial logistic regression of BRF trajectory class membership on education, adjusted for sex, age, marital status, and self-rated health. The analysis revealed several significant differences between trajectory classes after Bonferroni-Holm correction. Participants with ≥ 12 years of schooling compared to those with ≤ 11 years were more likely to be assigned to class A (*Maintaining low-to-moderate alcohol consumption and an otherwise healthy lifestyle*) compared to class D (*Maintaining low-to-moderate or high alcohol consumption and daily smoking*; *p* < 0.001), class E (*Maintaining low-to-moderate or high alcohol consumption*,* daily smoking*,* and (gaining) overweight/obesity*; *p* < 0.001), class H (*Maintaining smoking and overweight/obesity*; *p* < 0.001), class F (*Maintaining alcohol consumption and obesity*; *p* = 0.002), or class G (*Maintaining absence of BRFs*; *p* < 0.001). Similarly, they were more likely to be in class B (*Maintaining alcohol consumption and (gaining) overweight*) than in classes D (*p* < 0.001), E (*p* < 0.001), or H (*p* = 0.001). They were also more likely to belong to class C (*Maintaining high alcohol consumption and (achieving) non-smoking*) compared to classes D (*p* < 0.001), E (*p* < 0.001), or H (*p* = 0.001).

**Table 4:** Association between education and trajectories of co-occurring behavioral health risk factors

	Class A		Class B		Class C		Class D		Class E		Class F		Class G	
Reference	OR	95% CI	OR	95% CI	OR	95% CI	OR	95% CI	OR	95% CI	OR	95% CI	OR	95% CI
Class B	1.72 ^a^	0.98–3.02												
Class C	1.22	0.47–3.16	0.71	0.28–1.76										
Class D	9.29 ^c^	4.79–18.02	5.40 ^c^	2.82–10.36	7.65 ^c^	2.82–20.72								
Class E	7.41 ^c^	3.70–14.82	4.30 ^c^	2.14–8.67	6.10 ^c^	2.36–15.71	0.80	0.36–1.75						
Class F	3.78 ^b^	1.81–7.90	1.92 ^a^	0.88–4.20	2.70 ^b^	0.94–7.90	0.36 ^b^	0.15–0.83	0.45 ^a^	0.18–1.09				
Class G	3.89 ^b^	1.88–8.04	2.17 ^a^	1.04–4.63	3.13 ^b^	1.14–8.53	0.41 ^a^	0.18–0.91	0.51 ^a^	0.22–1.18	1.04	0.43–2.56		
Class H	7.60 ^c^	3.15–18.36	4.42 ^c^	1.83–10.64	6.26 ^c^	2.06–18.96	0.82	0.32–2.01	1.03	0.39–2.67	2.29 ^a^	0.82–6.41	2.01 ^a^	0.73–5.53

Class A – Maintaining low-to-moderate alcohol consumption and an otherwise healthy lifestyle

Class B – Maintaining alcohol consumption and (gaining) overweight

Class C – Maintaining high alcohol consumption and (achieving) non-smoking

Class D – Maintaining low-to-moderate or high alcohol consumption and daily smoking

Class E – Maintaining low-to-moderate or high alcohol consumption, daily smoking, and (gaining) overweight/obesity

Class F – Maintaining alcohol consumption and obesity

Class G – Maintaining absence of BRFs

Class H – Maintaining smoking and overweight/obesity

*OR* odds ratio, *CI* confidence interval. Education: 0, ≤ 11 years of schooling (reference); 1, ≥ 12 years of schooling. Adjusted for sex, age, marital status, and self-reported health. Bold values indicate statistical significance after Bonferroni-Holm correction for multiple comparisons. Odds ratios of 1.44/0.70, 2.48/0.40 and 4.27/0.23 were used as benchmarks for quantifying small (^a^), medium (^b^), and large (^c^) effects, corresponding to Cohen’s d values of ≥ 0.2, ≥ 0.5, and ≥ 0.8, respectively.

Additionally, several non-significant differences showed meaningful effect sizes, indicating potentially important patterns that may warrant attention despite not reaching statistical significance. For example, participants with ≥ 12 years of schooling compared to those with ≤ 11 years were more likely to be assigned to class G (*Maintaining absence of BRFs*) than to class E (*Maintaining low-to-moderate or high alcohol consumption*,* daily smoking*,* and (gaining) overweight/obesity*), and less likely to be in class G compared to classes B (*Maintaining alcohol consumption and (gaining) overweight*) and C (*Maintaining high alcohol consumption and (achieving) non-smoking*).

## Discussion

This study offers insights into trajectories of co-occurring alcohol consumption, tobacco smoking, overweight, and physical inactivity and their association with education in an adult general population sample. Two main findings emerged. First, eight distinct trajectories of co-occurring BRFs were identified, with the largest group of persons being characterized by a consistently healthy lifestyle pattern, except for low-to-moderate alcohol consumption. Second, persons with higher education were more likely to belong to trajectory classes characterized by a combination of alcohol consumption and non-smoking as well as with favorable BRF changes over time. Persons with lower education were more likely to belong to classes with multiple BRFs, particularly those involving tobacco smoking, along with unfavorable changes over time.

The data clearly revealed heterogeneity in the development of multiple co-occurring BRFs over time, with the best representation provided by eight latent trajectory classes. There are groups of persons for whom some BRF trajectories are clustered more than others. The most prevalent combination of BRFs was alcohol consumption and overweight or obesity, found in one-third of participants, followed by alcohol consumption and tobacco smoking, present in one-fifth of participants. The link between alcohol consumption and body mass index is plausible, given the high prevalence of overweight/obesity and alcohol consumption in the German general population [37, 38]. Alcohol may contribute to weight gain due to its high caloric density and potential appetite-stimulating effect [39]. Similarly, the co-occurrence and mutually reinforcing dynamics of alcohol and tobacco consumption have been demonstrated in numerous studies [40, 41]. This combination of BRFs appears to be particularly relevant to disease and premature death. For example, evidence suggests that, when present in combination, alcohol and tobacco consumption have multiplicative or greater effects on the risk of head and neck cancer [42].

Alcohol consumption was a common risk factor in the majority of the BRF trajectories and showed a considerable stability over time. Six of the eight identified trajectories involved stable alcohol consumption, either at a low-to-moderate or high level. In our sample, the most common BRF trajectory was stable low-to-moderate alcohol consumption and an otherwise healthy lifestyle. This is in line with data from a study of U.S. adults [14]. Notably, this group of persons would have been considered demonstrating an overall healthy lifestyle just a few years ago, as low-to-moderate alcohol consumption was, and still is, considered acceptable in many parts of society. One consequence is the paucity of brief behavior change interventions that consider them when motivating people to reduce their alcohol consumption. Meanwhile, growing evidence suggests that alcohol consumption has no beneficial health effect compared to abstinence, with even low levels linked to an increased risk of chronic disease and premature death [43–46]. In a country with high per capita consumption, alcohol consumption remains a major risk factor in a wide range of BRF patterns. This finding suggests that behavior change interventions should definitely focus on motivating persons to reduce their alcohol consumption. In our sample of adults reporting alcohol consumption in the previous year, two small classes emerged with high probabilities of current alcohol abstinence over time, together accounting for nearly 10% of the sample. One trajectory class was characterized by an overall healthy lifestyle, while the other showed high probabilities of tobacco smoking and overweight or obesity. These findings show that persons who report being currently abstinent may still be exposed to other BRFs and/or may either be former drinkers or consume alcohol very rarely. This supports the need for interventions targeting multiple BRFs simultaneously, rather than focusing solely on alcohol consumption. It also underscores the importance of accurately defining and measuring alcohol abstinence to avoid biased conclusions when investigating the health effects of alcohol consumption [47].

Surprisingly, physical inactivity played virtually no role in differentiating the BRF trajectories. In contrast to other general population samples [1], participants in our study reported high and even increasing engagement in MVPA. While the large student population in the city where the sample had been drawn, its cycling infrastructure, and access to nature may have contributed to higher activity levels, it is likely that MVPA was overestimated, which is a common issue with self-reported physical activity data [48]. The observed increase in MVPA over time may be linked to the weight gain observed in most classes, as persons who gain weight may respond by increasing their activity levels. It may also be that persons with weight gain are already out of breath from less intense activities and have not consciously increased their physical activity.

Educational disparities in BRF trajectories were most pronounced for multi-risk combinations involving tobacco smoking, as reflected by the largest odds ratios. Compared to persons with higher education, those with lower education were more likely to be classified into classes characterized by multiple co-occurring BRFs, particularly when these included tobacco smoking and unfavorable BRF changes over time. The highest proportions of persons with lower education were observed in class E (*Maintaining low-to-moderate or high alcohol consumption*,* daily smoking*,* and (gaining) overweight/obesity*), followed by class H (*Maintaining smoking and overweight/obesity*) and class D (*Maintaining low-to-moderate or high alcohol consumption and daily smoking*). This finding supports data from a survey of the general population in Germany, suggesting that tobacco smoking and overweight are particularly prevalent in populations with lower education [1]. Tobacco smoking, in combination with alcohol consumption and excess weight, may be of particular importance for the development of disease and premature death [49], thereby contributing to health disparities. Overall, these findings align with the Health Lifestyle Theory, which posits a tendency for BRFs to cluster among persons with low socio-economic position [16]. Explanations include social norms that may encourage greater acceptance of these BRFs, along with limited access to health information and resources.

Although persons with higher education were more likely to belong to classes with fewer BRFs compared to those with lower education, our findings do not support previous research suggesting they are more likely to adopt a lifestyle free from all four BRFs [1]. Notably, the three classes with the highest proportions of persons with higher education all include alcohol consumption, either as a single BRF or combined with overweight, but not obesity. These were class A (*Maintaining low-to-moderate alcohol consumption and an otherwise healthy lifestyle*), class C (*Maintaining high alcohol consumption and (achieving) non-smoking*), and class B (*Maintaining alcohol consumption and (gaining) overweight*). The results might have been different if our sample had included persons who had been abstinent from alcohol for more than a year. However, it is also plausible that among highly educated persons, alcohol consumption is socially accepted and sometimes even encouraged, such as at business dinners or as part of social life [50]. It is important to keep in mind that Germany is a country with high per capita alcohol consumption [51]. Nevertheless, several studies suggest that high education is protective against alcohol-related harm and mortality [52].

Five limitations should be noted. First, although more than two-thirds of the target population were reached, selection bias is likely. The present study did not include persons who have been abstinent for longer than 12 months, and our findings may not be generalizable to countries with low per capita alcohol consumption. Furthermore, the sample predominantly represented persons with higher education. This is partly attributable to the socio-demographic structure of the Greifswald population, which includes a large proportion of university employees and students. Second, all measures used in this study were based on brief self-reports and may, as such, be subject to recall and response biases. However, self-reports enabled the reliable assessment of multiple BRFs over multiple timepoints in a large sample. Third, categorized variables were used. The categorization may not have captured nuances that are crucial for understanding BRF trajectories and their association with education. However, we aimed to align the categories with official recommendations by health organizations which can be particularly relevant for decision-makers when determining public health strategies. Fourth, we cannot rule out the possibility that the LCGA have led to over-extraction of classes. To minimize this risk, the number of random starts and final-stage optimizations was increased [53]. Additionally, several criteria were weighed up when deciding on the optimal number of classes. Fifth, although we used FIML, a standard approach for handling missing data in longitudinal modeling, it relies on the MAR assumption. In our sample, 456 participants completed all three follow-ups (55%), 125 completed two (15%), 116 completed one (14%), and 134 participated only at baseline (16%). This reduces the plausibility of the MAR assumption and may introduce bias if data were in fact missing not at random. However, MAR may be considered a reasonable assumption in multivariate datasets, as relationships between missingness and unobserved values can often be sufficiently accounted for by observed data. Moreover, previous research suggests that modest violations of the MAR assumption typically do not substantially alter the conclusions drawn from such analyses [54, 55].

## Conclusions

To conclude, this study demonstrates significant heterogeneity in the development of co-occurring BRFs over time and their complex association with education. Although persons with higher education were more likely to belong to trajectory classes with fewer BRFs and more favorable BRF changes over time, alcohol consumption remained a common risk factor in several of these trajectories. From a public health perspective, trajectories involving tobacco smoking combined with alcohol consumption, and excess weight, and unfavorable BRF changes over time, which disproportionately affect groups of persons with lower education, are particularly concerning. Our findings suggest that interventions, that intend to have population-wide and socially equitable impact, should address multiple BRFs and their socio-economic determinants rather than focusing on each BRF in isolation. It remains important to focus on reducing alcohol consumption within these interventions.

## Data Availability

The datasets analyzed during the current study are available from the corresponding author on reasonable request.

## Abbreviations

AIC: Information Criterion
BIC: Bayesian Information Criterion
BRF: Behavioral health Risk Factor
CI: Confidence Interval
LCGA: Latent Class Growth Analysis
MVPA: Moderate-to-Vigorous Physical Activity
OR: Odds Ratio
pr: Probability
SD: Standard Deviation
VLMR-LRT: Vuong-Lo-Mendell-Rubin adjusted Likelihood Ratio Test
